# Skeletal standardized uptake values obtained by quantitative SPECT/CT as an osteoblastic biomarker for the discrimination of active bone metastasis in prostate cancer

**DOI:** 10.1186/s41824-017-0006-y

**Published:** 2017-10-12

**Authors:** Ichiei Kuji, Tomohiko Yamane, Akira Seto, Yota Yasumizu, Suguru Shirotake, Masafumi Oyama

**Affiliations:** 1 Department of Nuclear Medicine, Saitama Medical University International Medical Center, 1397-1 Yamane, Hidaka, Saitama, 350-1298 Japan; 2 Department of Uro-Oncology, Saitama Medical University International Medical Center, 1397-1 Yamane, Hidaka, Saitama, 350-1298 Japan

**Keywords:** Bone metastasis, Quantitative SPECT/CT, SUV, Prostate cancer, ^99m^Tc-methylene-diphosphonate, CGZAS reconstruction

## Abstract

**Purpose:**

To investigate the improvement of prognostication of active bone metastatic burden by discriminating bone metastases from degenerative changes in hot foci, using skeletal standardized uptake values (SUVs) by quantitative bone single photon emission tomography/computed tomography (SPECT/CT) in patients with prostate cancer.

**Methods:**

We investigated 170 patients with prostate cancer who underwent skeletal quantitative SPECT/CT using ^99m^Tc-methylene-diphosphonate (MDP), through conjugate gradient reconstruction with tissue zoning, attenuation, and scatter corrections applied, called as CGZAS reconstruction, in a retrospective cohort study. The maximum, peak, and average SUVs (SUVmax, SUVpeak, and SUVave, respectively) were obtained for visually normal thoracic (T; *n* = 100) and lumbar (L; *n* = 140) vertebral bodies as controls, as well as for bone metastases (*n* = 126) and degenerative changes (*n* = 114) as hot foci. They were also correlated with age, body-weight, height, biochemistry data, and extent of disease (EOD). Discrimination accuracy of the SUVs for bone metastases in hot foci was evaluated by a patient-based and lesion-based receiver-operator characteristic curve (ROC) analysis.

**Results:**

The skeletal SUVmax was 7.58 ± 2.42 for T, 8.12 ± 12.24 for L, 16.73 ± 6.74 for degenerative changes, and 40.90 ± 33.46 for bone metastases. The SUVs of the bone metastasis group were significantly (*p* < 0.001) greater than of the other three groups. With disease extent, serum alkaline phosphatase and prostate specific antigen were increased, while SUVs for bone metastases were decreased in EOD grade 4. In ROC analyses for bone metastases by skeletal SUVs demonstrating the diagnostic accuracy of skeletal SUVs for discriminating bone metastasis from degenerative changes in hot foci, area under curves were 0.840, 0.817, and 0.845 in patient-based mode, and 0.932, 0.920, and 0.930 in lesion-based mode.

**Conclusions:**

The skeletal SUVs by ^99m^Tc-MDP SPECT/CT for active bone metastases were greater than those for degenerative changes in patients with prostate cancer, with a feasible discrimination accuracy in the hot foci. Therefore, skeletal SUVs, especially SUVmax, in quantitative bone SPECT/CT may be helpful indices for the prognostication of bone metastatic burden, improving discrimination of active bone osteoblastic metastases in patients with prostate cancer from frequently coexisting degenerative changes.

## Introduction

Prostate cancer is a common malignancy and the sixth most common cause of cancer-related mortality in the year 2015 among Japanese men (Miyoshi et al., 2015). Bone metastasis is seen in more than 90% of patients with metastatic castration-resistant prostate cancer (mCRPC) and is an important cause of death (Klaassen et al., 2017). Bone scintigraphy is a prevailing diagnostic test for the detection of bone metastasis in patients with prostate cancer because of its high sensitivity, cumulative evidence for detection, and easy accessibility (Langsteger et al., 2016). Analysis of the degree and extent of multiple bone metastases, cumulatively called as the extent of disease (EOD), has been based on visual diagnosis of whole-body images (Imai et al., 1992). Recent introduction of new therapies has focused on the subjective evaluation of the status of bone metastasis. For example, a semi-quantitative measurement of the bone scan index (BSI) by automated methods has been applied to investigate bone metastasis status (Nakajima et al., 2017). A single photon emission tomography/computed tomography (SPECT/CT) technique has been specifically employed in patients with prostate cancer (Helyar et al., 2010).

Presence off skeletal degenerative changes is frequent in prostate cancer, and may result in equivocal findings for bone metastases in whole-body scan (Zacho et al., 2015). In a differential diagnosis of bone metastasis from skeletal degenerative changes by skeletal SPECT/CT, localised skeletal destructive and/or sclerotic changes suggest bone metastases, while sclerotic changes or skeletal deformation along the marginal part of vertebra or joints suggest skeletal degenerative or inflammatory diseases (Tuncel et al., 2016). At an initial phase, bone metastases show limited skeletal anatomical changes that are detectable only by using bone SPECT; this is a more sensitive technique than the planar or whole-body scans. SPECT/CT has demonstrated additional diagnostic value to the standard skeletal scintigraphy (Helyar et al., 2010), and specifically, hybrid imaging using SPECT/CT has been proven to increase specificity and positive predictive value of the bone scintigraphy (Palmedo et al., 2014).

The standardized uptake value (SUV) is a feasible semi-quantitative parameter, frequently used for assessing uptake of radionuclide tracers. The SUV measurement in a modern SPECT/CT scan is reported to have adequate accuracy for quantitation of ^99m^Tc-radioactive concentration, with an error rate of 3.6% in phantom and 1.1% in patients with high activity bladder (Zeintl et al., 2010). To get better quantitative information from SPECT/CT scans, a high-resolution image reconstruction method with CT segmentation, termed as ordered subset conjugate-gradient minimizer (OSCGM) method (Armstrong & Hoffmann, 2016), and a conjugate gradient reconstruction with tissue zoning, attenuation, and scatter corrections applied (CGZAS) method have been introduced in commercial SPECT/CT devices (Gnesin et al., 2016).

It is not clear whether an SUV obtained by skeletal SPECT/CT scans and analysed using the CGZAS method has any clinical and diagnostic value for the evaluation of bone metastasis in patients with prostate cancer. Muzahir et al. reported that ^18^F–sodium fluoride (NaF) PET/CT was able to differentiate metastatic from degenerative joint disease by SUVmax in patients with CRPC (Muzahir et al., 2015). Similarly, the skeletal SUV by bone SPECT/CT has a potential to be a good biomarker of osteoblastic metabolism, although body size, renal function, and skeletal disease extent might affect its value. We hypothesised that precise skeletal SUVs measured by modern SPECT/CT can be used by itself to improve assessment of active bone metastases so that it can be demarcated from skeletal degenerative changes. Thus, the SUV obtained would reflect the aggressive osteoblastic activity in bone metastatic lesions, which is characteristic of metastasised prostate cancer.

The aim of this study was to clarify the clinical utility of skeletal SUVs for improving prognostication of active bone metastases, which were obtained by skeletal SPECT/CT scans and analysed using the CGZAS method. In particular, we investigated the ability of skeletal SUVs to discriminate bone metastases in patients with prostate cancer, and successfully demarcate active metastatic lesions from skeletal degenerative changes.

## Materials and methods

### Subjects

From a pool of 436 patients with prostate cancer who underwent bone scintigraphy in our hospital from 1 July 2015 to 30 May 2016, we retrospectively selected 170 male patients who also underwent skeletal quantitative SPECT/CT. Clinical indications to refer patients for bone scintigraphy in our hospital were at initial staging and restaging of patients with prostate cancer, and monitoring therapeutic effects in bone metastases in castration-resistant prostate cancer. For precise diagnosis, whole-body SPECT/CT scans were performed on cervical-to-thoracic and/or lumbar-to-pelvic segments, when any localised uptake in cranial, vertebral and pelvic bones was observed during the evaluation of the initial staging or restaging of the patients with prostate cancer (Helyar et al., 2010)

We selected patients from our database on the basis of data from the picture archiving and communication system. Inclusion criteria were: (1) first-time patients who underwent bone quantitative SPECT/CT because of suspected bone metastasis for completion of initial staging or restaging, usually showing more than intermediate risk (according to the National Comprehensive Cancer Network risk categorisation), indicated by either high serum prostate-specific antigen (PSA > 10 ng/mL) or high Gleason’s score (GS > 7) or clinical stage >T2b on prostate biopsy; and (2) follow-up patients who had already been diagnosed with bone metastases from prostate cancer and received therapies for re-evaluation of bone metastases.

All patients were positively diagnosed with prostate cancer by prostate biopsy. Subjects included 112 patients who underwent bone scintigraphy for the first time, as well as 58 follow-up patients who had confirmed bone metastases. From our medical records, we obtained basic profile data, including age, body-weight (BW), and height, as well as blood biochemistry data, including serum alkaline phosphatase (ALP), PSA, and serum creatinine (Cr), acquired at the same time or within 2 months (14.4 ± 14.0 days) of the SPEC/CT test. This retrospective cohort study was reviewed by our hospital institutional review board. Documentation of informed consent from each patient was waived as the study was observational and data were obtained as part of daily clinical practice.

### Bone SPECT/CT

The anterior and posterior whole-body scan and quantitative bone SPECT/CT co-registered/hybrid imaging was performed using a SPECT/CT scanner (Symbia Intevo, Siemens Healthcare K. K., Tokyo, Japan), 143 to 307 (average 204 ± 27) minutes after administration of 548 to 1170 (average 850.6 ± 168.1) MBq of ^99m^Tc-methylene diphosphonate (MDP; FUJIFILM RI Pharma, Co. Ltd., Tokyo, Japan). For acquiring whole-body images, SPECT/CT scanning was performed using the low-energy high-resolution collimator with a SPECT condition of continuous rotation at 6°, 10 s/view, 10 min/rotation, and a low-dose CT condition of 130 kV and 50 mAs with CARE-DOSE application (Siemens Healthcare K. K., Tokyo, Japan). The acquisition range of SPECT/CT scanning was thoracic and/or abdominal to the pelvic regions, collected by one or two scans. CT images were generated with a 5-mm slice thickness using a smooth reconstruction kernel. SPECT images were reconstructed using the CGZAS algorithm (xSPECT bone mode) based on CT zonal mapping with the conditions of iteration 48, subset 1, and a Gaussian filter of smoothness 5 mm. The reconstruction was performed with 2.54-mm cubic 256 × 256 matrices, integrating a scatter correction using dual-energy scatter window subtraction and an attenuation correction based on attenuation maps derived from CT data. To measure precise bone uptake, a CGZAS reconstruction with CT zonal mapping consisting of 5 zones, air, fat tissue, soft tissue, medullary bone, and bone cortex was used, and reconstruction was performed based on X-ray attenuation mapping (Gnesin et al., 2016). The spatial resolution of the skeletal SPECT images was improved by zonal mapping, especially at the margins of the bone. Regular calibration of the SPECT/CT system was performed with an internal ^57^Co point-source phantom. The SPECT reconstructed values were decay-corrected to the time of injection and final values of quantitative radioactivity concentration were obtained.

### Image interpretation

Three experienced physicians, who were certified by both the board of nuclear medicine and the board of diagnostic radiology, diagnosed skeletal lesions using a combination of SPECT and CT images. The findings of SPECT/CT were compared with the results of clinical follow-up every 3–6 months for 1 year after imaging, based on the consensus of the physicians as the gold standard. Clinical follow-up was performed by clinical examination, medical reports, imaging results, and PSA. Hot foci of degenerative changes were considered as skeletal degenerative diseases, such as osteophytes, degenerative intervertebral joint disease, degenerative tear of the marginal area of the vertebral body, or vertebral compression without sclerotic metastasis on CT images. Then, hot foci of bone metastases were identified in demarcated sclerotic bone metastatic areas, or in areas that could not be explained by the presence of degenerative changes. Skeletal disease extent was evaluated based on whole-body images according to literature, and scored on a 5-point scale, ranging from 0 to 4, to quantify the extent of disease (EOD) (Soloway et al., 1988).

### Image analysis and quantification

Quantitative measurement was performed on a Syngo Via workstation (Siemens Healthcare K. K., Tokyo, Japan) by an experienced physician specializing in nuclear medicine. SUV based on BW was calculated according to the equation given below; relative weight in voxels of interest (VOI) was assumed to be 1 g per 1 cm^3^.

$$ SUV=\frac{\left( concentration of radioactivity in\  VOI\right)/\left( volume of\  VOI\right)}{\left( injected radioactivity\right)/\left( body weight\right)} $$

We inspected and selected up-to 6 lesions from the visually hottest spots on SPECT images. The actual hottest 3 lesions were defined by comparison of SUVmax for bone metastases. SPECT/CT images were analysed and each spherical VOI area on the lesion for SUV measurement was set manually on each hot spot, ensuring that the whole area of each hot location was included. Other hot activity areas, such as renal pelvis, ureteral tracts, and bladder were carefully excluded. Diagnosis of each skeletal lesion detected as a hot spot was based on CT scans of the bone window. Skeletal hot lesions were divided into two groups; those diagnosed as signifying bone metastases and those that were caused by degenerative changes. The three hottest spots of bone metastasis or the hottest spot of degenerative changes for each patient were included in further analysis. One visually normal thoracic (T) and/or lumbar (L) vertebral body in each patient was defined as a control. Each spherical VOI was set on the T and/or L vertebral body without hot spots when possible, and were size-matched in each case.

The maximum, peak, and average SUVs (SUVmax, SUVpeak, and SUVave, respectively) of ^99m^Tc-MDP were obtained in visually normal vertebral bodies and skeletal lesions on SPECT/CT images based on the BW method. SUVpeak was defined as the average of the greatest value in a 1-cm sphere in the VOI. In addition, each metabolic volume (MV) and SUVave was obtained as the volume and average SUV at 40% threshold of the SUVmax in the bone metastases and degenerative changes groups. The three of the hottest lesions in the bone metastases group and the hottest lesion in the degenerative changes group were selected for further analysis according to the SUV measurement. If the patient showed no obvious abnormal uptake, the SUV in a normal vertebral body was allocated to the degenerative changes group.

### Comparison of SUVs between the bone metastases and degenerative changes groups

In a patient-based analysis, the highest SUV in the bone metastases and degenerative changes groups for each patient was included for comparison. Each SUV in the bone metastasis group was compared with that in the degenerative changes group. In a lesion-based analysis, analysed lesions included up-to three of the highest SUVs for each patient in the bone metastasis group, the highest SUVs for each patient in the degenerative changes group, and SUVs in all T and L.

### Evaluation of the effect of lesion size on SUVs

For evaluation of the effect of size on SUVs, bone metastasis and degenerative changes lesions were divided into 4 categories according to MV, as follows: <10, 10 to <20, 20 to <30, and ≥30 cm^3^. Each SUV from the bone metastases group was compared to an SUV from the degenerative changes group in the corresponding size category.

### Discrimination accuracy of SUVs for bone metastasis compared to degenerative changes in hot spots

Discrimination accuracy of each SUV in the bone metastases group in hot spots was compared with that in the degenerative changes group by a receiver-operator characteristic curve (ROC) analysis. In a patient-based analysis, ROC analyses were performed with and without the ALP and PSA parameters. In a lesion-based analysis, discrimination accuracy of each SUV for bone metastases versus degenerative changes groups was compared by ROC analysis.

### Sample number and statistical power planning

We assumed that the average SUVmax of bone metastatic lesions would be 10-fold greater than that of other lesions based on our preliminary small sample data (not published). The sample size was fixed by the statistical power analysis. Assuming that the size d, alpha, power, and allocation ratios of patients with bone metastasis to those without were 0.6, 0.05, 0.95, and 0.5, respectively, the required total sample size was estimated to be 166. Thus, we chose to include data from 170 subjects, which was slightly higher than the required number.

### Statistical analysis

Statistical analysis and graphing of data were performed using SPSS ver. 24.0 (IBM Corp.) and Prism 7.0b (GraphPad software, Inc.) software. Data distribution was analysed using the Shapiro-Wilk normality test. Data was expressed as average ± standard deviation. Differences between the SUVs of multiple groups were analysed using the Kruskal-Wallis test with Dunn’s multiple comparisons. The differences in basic profile data, blood biochemistry data, and SUVs, between the bone metastases and degenerative changes groups, were analysed by the Mann-Whitney U test. A *p* value of less than 0.05 was considered statistically significant for all the analyses. A box-and-whisker plot was constructed, with the bottom and top of the boxes representing the first and third quartiles, and the data minimum and maximum values as the ends of whiskers. The median was marked as a line in the box.

## Results

### Comparison of profile and blood data

The basic profile data and biochemical parameters of patients in the bone metastases and degenerative changes groups are shown in Table 1. There was no significant difference in fundamental profile data of age, body-weight, height, and Cr between the two groups (*p* = 0.424, 0.612, 0.063, and 0.333, respectively), though there were statistically significant differences in the biochemical parameters ALP and PSA (*p* = 0.001, each).

**Table 1:** Basic profile and blood biochemical data of patients included in the study

		n	mean	sd	*p*
Age (years-old)	BM	56	70.4	7.4	
	DC	114	71.3	6.8	
	Total	170	71.0	7.0	0.424
Body weight (kg)	BM	56	64.7	8.4	
	DC	114	63.8	9.9	
	Total	170	64.1	9.4	0.612
Height (cm)	BM	56	166.4	6.2	
	DC	114	164.4	6.5	
	Total	170	165.0	6.5	0.063
ALP (U/L)	BM	52	518.4	740.8	
	DC	101	238.2	89.0	
	Total	153	333.5	455.0	0.001**
PSA (ng/mL)	BM	53	278.7	1036.2	
	DC	106	92.7	791.7	
	Total	159	154.7	881.9	0.001**
Cr (mg/dL)	BM	53	0.96	0.77	
	DC	105	1.04	1.31	
	Total	158	1.01	1.16	0.333

*n* number, *sd* standard deviation, *BM* bone metastases, *DC* degenerative changes, *ALP* alkaline phosphatase, *PSA* prostate specific antigen, *Cr* creatinine, **p < 0.01; *p < 0.05

### Representative images

**Fig. 1:**
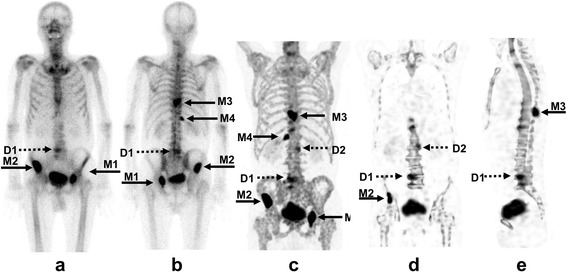
A representative whole-body (WB) image (**a**, **b**), a maximum intensity projections (MIP) image (**c**), and coronal and sagittal SPECT images (**d**, **e**) in the same standardized uptake value (SUV) window range (0–15). Note the enhanced contrast of uptake in regions of bone metastases (solid arrows) compared to regions of degenerative changes (dotted arrows). Lesions indicated as M1 to M4 were bone metastases, and those indicated as D1 and D2 were degenerative changes. Corresponding SUVmax measurements were M1, 117.9; M2, 90.80; M3, 50.90; M4, 39.90; D1, 24.08; D2, 13.26, and MV measurements were M1, 8.87; M2, 19.35; M3, 7.25; M4, 2.73; D1, 15.80; and D2, 9.18, respectively

Representative SPECT images and SUVs in a patient with both bone metastases and degenerative changes are shown in Fig. 1. Active bone metastases exhibited greater SUVs than degenerative changes, irrespective of MVs, showing the difficulty in discriminating the lesions in whole-body images.

### Comparison of image quantitative parameters

**Fig. 2:**
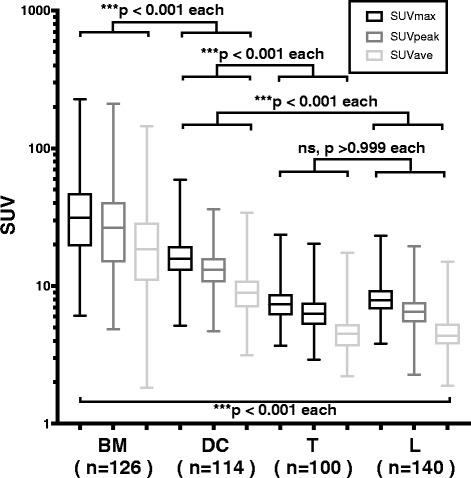
Summary of skeletal standardized uptake values (SUVs; SUVmax, maximum of SUV; SUVpeak, peak of SUV; SUVave, average of SUV) in each group. All SUVs in bone metastases (BM) group were significantly greater than in degenerative changes (DC) and thoracic (T) and lumbar (L) groups. Please note that the SUVs scale is logarithmic

The SUVs of the bone metastases group were significantly *(p <* 0.001) greater than those in the other three groups, as shown in Fig. 2. In particular, each SUV of the lesions in the bone metastases groups was consistently and significantly greater than the SUVs of the lesions in the degenerative changes group. A summary of the SUVs of the degenerative changes and bone metastases groups is shown in Table 2. However, the MV of the lesions in the bone metastases group was significantly lesser than the MV of the degenerative changes group. This may be explained by the fact that the VOI in the lesions with higher SUV tended to be defined smaller by the higher threshold.

**Table 2:** Summary of SUV measurements in the DC and BM groups

		Total			DC			BM			
		n	mean	sd	n	mean	sd	n	mean	sd	*p*
T		100			75			25			
	SUVmax		7.58	2.42		7.52	2.63		7.76	1.69	0.242
	SUVpeak		6.51	2.12		6.49	2.30		6.59	1.48	0.399
	SUVave		4.62	1.68		4.61	1.86		4.67	0.93	0.292
L		140			101			39			
	SUVmax		8.12	2.24		7.95	2.37		8.56	1.80	0.020*
	SUVpeak		6.68	1.93		6.57	2.01		6.97	1.72	0.083
	SUVave		4.54	1.38		4.46	1.47		4.76	1.06	0.017*
Lesions		240			114			126			
	SUVmax		29.42	27.45		16.73	6.74		40.90	33.46	<0.001***
	SUVpeak		24.50	24.14		13.74	4.52		34.24	29.91	<0.001***
	SUVave		17.41	17.30		9.47	3.94		24.59	21.19	<0.001***
	MV		23.99	26.16		26.27	26.21		21.92	26.04	0.009**

*n* number, *sd* standard deviation, *T* thoracic vertebral body, *L* lumbar vertebral body, *BM* bone metastases, *DC* degenerative changes, ***p < 0.001; **p < 0.01; *p < 0.05

### Effects of the MV size category on difference in SUVs

**Fig. 3:**
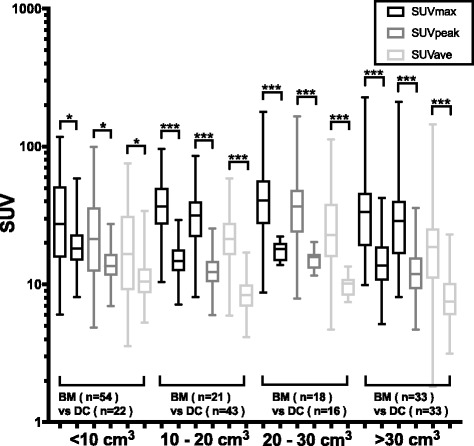
SUVs in areas of bone metastases (BM) and those in areas of degenerative changes (DC) in the MV size categories represented as box-and-whisker plots. The SUVs showed significant differences between BM and DC in all MV categories. Please note that the SUV scale is logarithmic. ***p < 0.001; *p < 0.05

Figure 3 shows a comparative analysis of differences between SUVs of the bone metastases and degenerative changes groups in various MV size categories. There were significant differences between the SUVs of the two groups in all MV size categories. In particular, all the SUV parameters differed significantly (*p* < 0.001) between the bone metastases and degenerative changes groups in the three categories, with MV values of greater than 20 cm^3^ and less than 10 cm^3^. The differences in the SUVmax, SUVpeak, and SUVave were significant with *p*-value of 0.021, 0.026, and 0.021, respectively. Thus, the ability of SUVs to discriminate bone metastases and degenerative changes was effective in all size categories.

### Correlation of SUVs with EOD

**Fig. 4:**
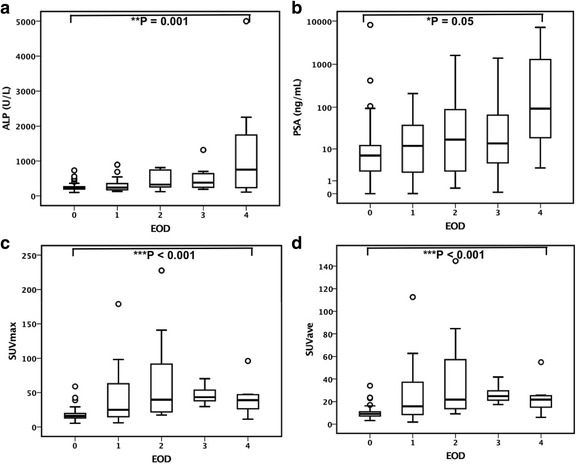
Changes in analysis parameters with skeletal disease extent. There was significant change in alkaline phosphatase (ALP, **a**), prostate specific antigen (PSA, **b**), maximum and average of standardized uptake values (SUVmax, **c**; SUVave, **d**) with change in extent of disease (EOD) grade. ALP and PSA demonstrated an increasing trend with increase in EOD grade, while SUVmax showed a relatively lower maximum value in grade 4 disease. Please note that the scale of the graph for PSA (**b**) is logarithmic

The number of subjects included and details of inclusion status in relation to the EOD grades are shown in Table 3. The biochemical blood parameters ALP and PSA, as well as the SUVs correlated significantly with the EOD grades (Fig. 4). There was a trend of increasing ALP and PSA with higher EOD. In contrast, SUVs for the bone metastases increased with higher EOD from grade 1 to grade 3, while the SUV was reduced in patients with grade 4 disease.

**Table 3:** Number of subjects and details of inclusion status in relation to extent of disease (EOD) grades

		EOD grades					Total
		0	1	2	3	4	
Status	Initial	88	15	3	3	3	112
	Follow-up	26	13	8	6	5	58
Total		114	28	11	9	8	170

### Discrimination accuracy of SUVs for active bone metastases

**Fig. 5:**
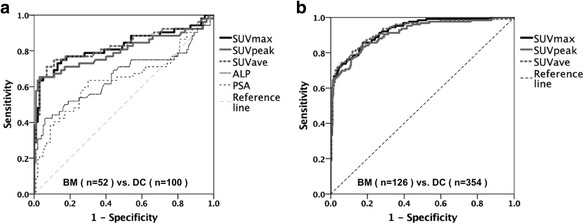
Receiver-operator characteristic curve (ROC) analyses of the discrimination efficacy for bone metastatic (BM) versus degenerative change (DC) lesions. Skeletal standardized uptake values (SUVs; SUVmax: maximum of SUV; SUVpeak: peak of SUV; SUVave: average of SUV), alkaline phosphatase (ALP) and prostate specific antigen (PSA) in patient-based (**a**) and lesion-based (**b**) analyses have been plotted

Results of patient-based and lesion-based ROC curve analyses between groups are shown in Fig. 5. The area under the curve (AUC) values of the SUVave and SUVmax curves were greater than that of the SUVpeak curve. The AUC values for SUVave and SUVmax were similar for both the patient-based and lesion-based analyses. SUVs had greater accuracy for discriminating active bone metastasis in the patient-based analysis than serum ALP and PSA (Table 4). Hot foci in bone SPECT/CT may be discriminated using SUVs with a certain accuracy; AUCs of the SUVpeak, SUVmax, and SUVave were 0.840, 0.817, and 0.845 in the patient-based mode, and 0.932, 0.920, and 0.930 in the lesion-based mode, respectively.

**Table 4:** Results of receiver-operator characteristic curve analyses of bone metastases from patient-based and lesion-based analyses

	AUC	Confidence interval	
		lower limit	upper limit
Patient-based with ALP and PSA (52 BM vs. 100 DC)			
SUVmax	0.824	0.742	0.906
SUVpeak	0.801	0.712	0.889
SUVave	0.829	0.748	0.911
ALP	0.660	0.557	0.763
PSA	0.640	0.538	0.742
Patient-based (56 BM vs. 114 DC)			
SUVmax	0.808	0.723	0.893
SUVpeak	0.785	0.694	0.875
SUVave	0.813	0.729	0.898
Lesion-based (126 BM vs. 354 DC)			
SUVmax	0.926	0.900	0.953
SUVpeak	0.911	0.879	0.943
SUVave	0.924	0.895	0.954

*AUC* area under curve, *BM* bone metastases, *DC* degenerative changes, *ALP* alkaline phosphatase, *PSA* prostate specific antigen

## Discussion

In the present study, we prove that a discrimination of active bone metastasis can be established with high accuracy in patients with prostate cancer based on SUV parameters. Thus, skeletal SUVs may help the differentiation of active bone metastasis from degenerative changes in hot foci in patients with prostate cancer. A pathological osteoblastic nature of bone metastases from prostate cancer is derived by the complex molecular interactions behind prostate cancer, affecting the balanced activity of osteoclasts and osteoblasts (Florimonte et al., 2016). During aging, the inflammation and tissue remodelling in chondral tissue around bone leads to calcification and ossification (Hayes et al., 2013). The different osteoblastic mechanism may affect SUV in prostate cancer with bone metastases and degenerative changes, reflecting the pathological osteoblastic nature of prostate cancer activity in higher SUV.

We observed that the skeletal SUVmax and SUVave in visually normal vertebral bodies in patients with prostate cancer were relatively low, at 7.58 ± 2.42 and 4.62 ± 1.68 in T, and 8.12 ± 2.24 and 4.54 ± 1.38 in L regions, respectively. Our observations are in agreement with previous publications reporting a SUVmax of 7.1 ± 0.4 (Kaneta et al., 2016), or a SUVave of 5.91 ± 1.54 (Cachovan et al., 2013) in normal vertebral bodies. In our study, the SUVs in regions with degenerative changes were greater than in visually normal vertebral bodies; however, the SUVs pertaining to degenerative changes were usually much lower than the skeletal SUVs in active bone metastases. This may be primarily due to the aggressive osteoblastic characteristic of bone metastases in prostate cancer. We speculate that the re-declined trend in SUVs with EOD grade 4 as high bone metastatic tumour burden is due to the decrease in the binding concentration of bone agents; this decrease is caused by the relative decrease in the amount of ^99m^Tc-MDP binding to the skeletal lesions, when the mass of bone metastasis increases due to disease extent to grade 4. In the phase 3, double-blind, randomised ALSYMPCA trial, relative risk for first symptomatic skeletal event was higher in EOD grade 3 than that in EOD grade 1 (Sartor et al., 2014). This phenomenon might account for the reason why nuclear medicine therapy by bone seeking radiopharmaceuticals was less effective in patients with EOD grade 4, in addition to increased tumour burden and its advanced stage (Kraeber-Bodere et al., 2000).

We observed that SUV correlated with disease diagnosis of bone metastasis even in cases with MV less than 10 cm^3^. In this study, the modern SPECT/CT system demonstrated visually effective and quantitative images than those reported by ordinal SPECT/CT (Beck et al., 2016). CT attenuation correction with CT zone mapping of bone cortex and medulla gave us the precise localisation of activity coming from the CT marginal resolution. A standard chi-square minimisation method underestimated the mean counts drawn from Poisson distribution. To remedy this, we used the CGZAS method and improved the estimation by using a region-weighted chi-square minimisation method proposed by Mighell (Mighell, 1999). Gnesin et al. concluded in their publication that this system provided accurate activity recovery within 10% of correct values and its performances were comparable to current generation PET/CT systems (Gnesin et al., 2016). Thus, image reconstruction by the CGZAS method improved both visual and quantitative analysis of SPECT/CT images compared with reconstruction by an ordinal OSCGM method (Armstrong & Hoffmann, 2016). To our best knowledge, this is the first report about clinical application and validation of the CGZAS method in patients with prostate cancer.

Our results suggest that the skeletal SUV can function as a reliable osteoblastic biomarker for discriminating active bone metastases with feasible accuracy. In recent literature, where bone scintigraphy was applied as a diagnostic tool, the BSI was proposed to be a good index for the extent of bone metastasis (Dennis et al., 2012; Takahashi et al., 2012; Ulmert et al., 2012; Nakajima et al., 2013; Kaboteh et al., 2013). An automated analysis using BSI has shown higher accuracy of 0.957 in AUC than the accuracy of our AUCs by the SUVs in prostate cancer on a per patient basis, using a specialized Japanese database (Nakajima et al., 2013). Since the automated methods with BSI analysis also depended on data from a database discriminating high-risk lesions from ordinal bone scans, it was generally impossible to detect bone metabolic disease. Compared with BSI, SUV seems to be a more sensitive index with a wider dynamic range, reflecting osteoblastic activity for each skeletal lesion with a suitable threshold; this is because SUV was derived from precise 3-dimensional data, while BSI was derived from 2-dimensional data.

Since SUV reflects active osteoblastic nature, it might be useful for the classification of skeletal lesions to diagnose biological aspects and select suitable therapy. SUV could be applicable to the dosimetry of bone lesions by radionuclide bone-targeted therapy with bone-seeking agents, since the concentration of the bone-seeking agents would be expected to be parallel to SUV (Pacilio et al., 2016). In addition to monitoring the effect of radionuclide bone-targeted therapy, including ^89^Sr-chloride and ^223^Ra-dichloride that are available in Japan, SUV might be a direct index enabling the prediction of an effect in each bone metastasis based on each radiation dose, as well as of a side-effect on the haematopoietic activity by estimating the radiation dose in the bone marrow (Florimonte et al., 2016; Kraeber-Bodere et al., 2000; Miederer et al., 2015; Alva et al., 2017).

In the current study, the SUVmax by NaF-PET/CT in degenerative joint diseases was 2.6–49.9 (mean, 6.2), which was less than the bone SUVmax (mean, 16.7) by bone SPECT/CT. In contrast, the SUVmax by NaF-PET/CT in bone metastases was 11.2–188 (mean, 160), which was higher than the SUVmax (mean, 40.9) by bone SPECT/CT (Muzahir et al., 2015). AUC of 0.964 by NaF-PET/CT was slightly higher, but comparable to AUC of 0.926 observed in the present study, after lesion-based ROC analysis. As a result, NaF-PET/CT data was 2-fold higher uptake and high target-to-background ration than ^99m^Tc-MDP due to faster blood clearance caused by lower protein binding and lower molecular weight (Langsteger et al., 2016). Clinical investigation using bone SUV of ^99m^Tc-biphosphonate analogs is rare. Thus, since SUVmax gathered using the NaF-PET technique has been reported to be helpful in assessing tumour burden and response to therapy, as well as in predicting outcome in bone metastases, skeletal bone SPECT/CT may serve as an effective surrogate for NaF-PET (Cook et al., 2011; Rohren et al., 2015). It is also well-known that the SPECT/CT method using radiopharmaceuticals with a longer half-life is cost-effective, with greater insurance coverage and reimbursement, and thus, more accessible to patients and physicians. In addition, an innovative development of detector might set high-resolution bone SPECT/CT method comparable to NaF-PET in the future (Langsteger et al., 2016). As an advantage, 3-min SPECT/CT has been reported to be sufficient for the assessment of bone metastases as add-on to planar scintigraphy (Zacho et al., 2017). Lastly, it has been demonstrated that whole-body SPECT/CT may be more accurate and cost-effective than whole-body planar plus SPECT/CT (McLoughlin et al., 2016).

This study suffered from several limitations, including the lack of scans of the chest or lumbar-pelvic portion in several SPECT/CT images. Even with the availability of whole-body scans for reference, there is a possibility that lesions with the highest uptake may have accidentally been excluded our analyses. Additionally, we omitted patients with proven bone metastases who did not undergo quantitative SPECT/CT scans. Therefore, there is a possibility of selection bias in our study.

## Conclusion

The skeletal SUVs by ^99m^Tc-MDP of bone metastases were greater than that of degenerative changes in patients with prostate cancer with adequate discrimination accuracy in the hot foci. Skeletal SUVs in quantitative SPECT/CT may be useful for the prognostication of bone osteoblastic metastatic burden in patients with prostate cancer. As the most accessible and easily measured bone SUV parameter, SUVmax might be the best candidate to serve as an osteoblastic biomarker for skeletal diseases in daily clinical practice.

## References

[CR1] Alva A, Nordquist L, Daignault S, George S, Ramos J, Albany C (2017). Clinical correlates of benefit from Radium-223 therapy in metastatic castration resistant prostate cancer. Prostate.

[CR2] Armstrong IS, Hoffmann SA (2016). Activity concentration measurements using a conjugate gradient (Siemens xSPECT) reconstruction algorithm in SPECT/CT. Nucl Med Commun.

[CR3] Beck M, Sanders JC, Ritt P, Reinfelder J, Kuwert T (2016). Longitudinal analysis of bone metabolism using SPECT/CT and ^99m^Tc-diphosphono-propanedicarboxylic acid: comparison of visual and quantitative analysis. EJNMMI Res.

[CR4] Cachovan M, Vija AH, Hornegger J, Kuwert T (2013). Quantification of ^99m^Tc-DPD concentration in the lumbar spine with SPECT/CT. EJNMMI Res.

[CR5] Cook G, Parker C, Chua S, Johnson B, Aksnes AK, Lewington VJ (2011). ^18^F-fluoride PET: changes in uptake as a method to assess response in bone metastases from castrate-resistant prostate cancer patients treated with ^223^Ra-chloride (Alpharadin). EJNMMI Res.

[CR6] Dennis ER, Jia X, Mezheritskiy IS, Stephenson RD, Schoder H, Fox JJ (2012). Bone scan index: a quantitative treatment response biomarker for castration-resistant metastatic prostate cancer. J Clin Oncol.

[CR7] Florimonte L, Dellavedova L, Maffioli LS (2016). Radium-223 dichloride in clinical practice: a review. Eur J Nucl Med Mol Imaging.

[CR8] Gnesin S, Leite Ferreira P, Malterre J, Laub P, Prior JO, Verdun FR (2016). Phantom validation of Tc-99m absolute quantification in a SPECT/CT commercial device. Comput Math Methods Med.

[CR9] Hayes AJ, Reynolds S, Nowell MA, Meakin LB, Habicher J, Ledin J (2013). Spinal deformity in aged zebrafish is accompanied by degenerative changes to their vertebrae that resemble osteoarthritis. PLoS One.

[CR10] Helyar V, Mohan HK, Barwick T, Livieratos L, Gnanasegaran G, Clarke SE (2010). The added value of multislice SPECT/CT in patients with equivocal bony metastasis from carcinoma of the prostate. Eur J Nucl Med Mol Imaging.

[CR11] Imai K, Tomaru Y, Ohnuki T, Yamanaka H, Sakai H, Kanetake H (1992). Significance of a new stratification of alkaline phosphatase and extent of disease in patients with prostate carcinoma with bone metastasis. Cancer.

[CR12] Kaboteh R, Gjertsson P, Leek H, Lomsky M, Ohlsson M, Sjostrand K (2013). Progression of bone metastases in patients with prostate cancer - automated detection of new lesions and calculation of bone scan index. EJNMMI Res.

[CR13] Kaneta T, Ogawa M, Daisaki H, Nawata S, Yoshida K, Inoue T (2016). SUV measurement of normal vertebrae using SPECT/CT with Tc-99m methylene diphosphonate. Am J Nucl Med Mol Imaging.

[CR14] Klaassen Z, Howard LE, de Hoedt A, Amling CL, Aronson WJ, Cooperberg MR (2017). Factors predicting skeletal-related events in patients with bone metastatic castration-resistant prostate cancer. Cancer.

[CR15] Kraeber-Bodere F, Campion L, Rousseau C, Bourdin S, Chatal JF, Resche I (2000). Treatment of bone metastases of prostate cancer with strontium-89 chloride: efficacy in relation to the degree of bone involvement. Eur J Nucl Med.

[CR16] Langsteger W, Rezaee A, Pirich C, Beheshti M (2016). ^18^F-NaF-PET/CT and ^99m^Tc-MDP bone Scintigraphy in the detection of bone metastases in prostate cancer. Semin Nucl Med.

[CR17] McLoughlin LC, O'Kelly F, O'Brien C, Sheikh M, Feeney J, Torreggiani W (2016). The improved accuracy of planar bone scintigraphy by adding single photon emission computed tomography (SPECT-CT) to detect skeletal metastases from prostate cancer. Ir J Med Sci.

[CR18] Miederer M, Thomas C, Beck J, Hampel C, Krieger C, Baque PE (2015). Haematopoietic toxicity of radium-223 in patients with high skeletal tumour burden. Nuklearmedizin.

[CR19] Mighell K (1999). Parameter estimation in astronomy with Poisson-distributed data. I. The *X*^*2*^*r* statistic. Astrophys J.

[CR20] Miyoshi Y, Noguchi K, Yanagisawa M, Taguri M, Morita S, Ikeda I (2015). Nomogram for overall survival of Japanese patients with bone-metastatic prostate cancer. BMC Cancer.

[CR21] Muzahir S, Jeraj R, Liu G, Hall LT, Rio AM, Perk T (2015). Differentiation of metastatic vs degenerative joint disease using semi-quantitative analysis with ^18^F-NaF PET/CT in castrate resistant prostate cancer patients. Am J Nucl Med Mol Imaging..

[CR22] Nakajima K, Edenbrandt L, Mizokami A (2017) Bone scan index: a new biomarker of bone metastasis in patients with prostate cancer. Int J Urol. 10.1111/iju.13386 10.1111/iju.13386 28556293

[CR23] Nakajima K, Nakajima Y, Horikoshi H, Ueno M, Wakabayashi H, Shiga T (2013). Enhanced diagnostic accuracy for quantitative bone scan using an artificial neural network system: a Japanese multi-center database project. EJNMMI Res.

[CR24] Pacilio M, Ventroni G, De Vincentis G, Cassano B, Pellegrini R, Di Castro E (2016). Dosimetry of bone metastases in targeted radionuclide therapy with alpha-emitting ^223^Ra-dichloride. Eur J Nucl Med Mol Imaging.

[CR25] Palmedo H, Marx C, Ebert A, Kreft B, Ko Y, Turler A (2014). Whole-body SPECT/CT for bone scintigraphy: diagnostic value and effect on patient management in oncological patients. Eur J Nucl Med Mol Imaging.

[CR26] Rohren EM, Etchebehere EC, Araujo JC, Hobbs BP, Swanston NM, Everding M (2015). Determination of skeletal tumor burden on ^18^F-fluoride PET/CT. J Nucl Med.

[CR27] Sartor O, Coleman R, Nilsson S, Heinrich D, Helle SI, O'Sullivan JM (2014). Effect of radium-223 dichloride on symptomatic skeletal events in patients with castration-resistant prostate cancer and bone metastases: results from a phase 3, double-blind, randomised trial. Lancet Oncol.

[CR28] Soloway MS, Hardeman SW, Hickey D, Raymond J, Todd B, Soloway S (1988). Stratification of patients with metastatic prostate cancer based on extent of disease on initial bone scan. Cancer.

[CR29] Takahashi Y, Yoshimura M, Suzuki K, Hashimoto T, Hirose H, Uchida K (2012). Assessment of bone scans in advanced prostate carcinoma using fully automated and semi-automated bone scan index methods. Ann Nucl Med.

[CR30] Tuncel M, Lay Ergun E, Caglar TM (2016). Clinical impact of SPECT-CT on bone scintigraphy in oncology: pattern approach. J BUON.

[CR31] Ulmert D, Kaboteh R, Fox JJ, Savage C, Evans MJ, Lilja H (2012). A novel automated platform for quantifying the extent of skeletal tumour involvement in prostate cancer patients using the bone scan index. Eur Urol.

[CR32] Zacho HD, Manresa JA, Mortensen JC, Bertelsen H, Petersen LJ (2015). Observer agreement and accuracy in the evaluation of bone scans in newly diagnosed prostate cancer. Nucl Med Commun.

[CR33] Zacho HD, Manresa JAB, Aleksyniene R, Ejlersen JA, Fledelius J, Bertelsen H (2017). Three-minute SPECT/CT is sufficient for the assessment of bone metastasis as add-on to planar bone scintigraphy: prospective head-to-head comparison to 11-min SPECT/CT. EJNMMI Res.

[CR34] Zeintl J, Vija AH, Yahil A, Hornegger J, Kuwert T (2010). Quantitative accuracy of clinical ^99m^Tc SPECT/CT using ordered-subset expectation maximization with 3-dimensional resolution recovery, attenuation, and scatter correction. J Nucl Med.

